# A Microfluidic PET-Based Electrochemical Glucose Sensor

**DOI:** 10.3390/mi13040552

**Published:** 2022-03-30

**Authors:** Linda Yang, Zheng Zhang, Xin Wang

**Affiliations:** 1 College of Mechanical and Aerospace Engineering, Jilin University, Changchun 130022, China; yangld19@mails.jlu.edu.cn (L.Y.); zhangzheng21@mails.jlu.edu.cn (Z.Z.); 2 Key Laboratory of Bionic Engineering, Ministry of Education, Jilin University, Changchun 130022, China

**Keywords:** glucose sensor, microfluidic, PET-based, electrochemical detection

## Abstract

Paper-based microfluidic sensors have gained increased attention in the field of analytical assays in recent years due to their self-driven nature, ease of preparation, high integration, low reagent consumption, and low cost. However, paper-based microfluidic sensors still have many deficiencies when it comes to the detection of some specific detectors such as blood glucose. For example, the processing procedure for microfluidic channels is tedious, the sensor electrodes are easily damaged by bending, and they can only be used as disposable products. To solve the above problems, a PET-based microfluidic sensor was proposed in this paper, the performance of which was tested with glucose as the target detector. The experimental results showed that the analytical performance of this sensor is comparable to that of existing commercial glucose meters. This work provides implications for the substrate selection of microfluidic chips for some biochemical analyses.

## 1. Introduction

Blood glucose is an important indicator in the measurement of glucose metabolism. Glucose in the human body undergoes a series of chemical reactions such as aerobic oxidation and anaerobic enzymes to provide the necessary energy and required substances for the various physiological activities of the body. Blood glucose monitoring refers to the regular monitoring of changes in human blood glucose levels, which is especially important for diabetic patients [1]. It can also help diabetic patients develop blood glucose abnormalities and to then adjust their lifestyle, diet, and activity patterns through blood glucose indicators to help patients with abnormal blood glucose improve their indicators as early as possible. In addition, blood glucose level monitoring can help to adjust the treatment plan, real-time monitoring of blood glucose level can also reduce the risk of diabetes complications, improve the effect of blood glucose control, and improve the quality of life [2].

With the rapid development of society, people’s quality of life have been greatly improved, which has led to an increasing number of people with diabetes in China and a trend of younger development [3]. Although diabetes does not pose an immediate threat to the life of the patient, it can easily lead to various complications and therefore requires the early detection of blood glucose levels and intervention with targeted treatment. Currently, studies have shown that early diagnosis of glucose metabolism disorders can slow down the development of diabetes and reduce mortality [4].

In terms of glucose detection methods, current blood glucose detection technologies mainly include optical methods and electrochemical methods. There are two main principles used in optical detection methods. The first includes optical techniques without fluorescent chromophores including optical coherence tomography, polarimetry, thermal infrared spectroscopy, photoacoustic spectroscopy, and Raman spectroscopy [5]. The second is an implantable optical sensor with a fluorescent chromophore that detects changes in glucose concentration indirectly with the help of the detection of other molecular contents during the oxidation reaction between glucose oxidase and glucose [6]. Colorimetric detection based on the first principle has been widely employed in point of care testing (POCT) because of its simplicity and compatibility with camera-phone-based telemedicine [7,8,9]. Electrochemical detection is a method of converting chemical signals into electrical signals to achieve detection of the target, which can be achieved by adjusting and controlling the potential and current of the working electrode, or by fixing specific nanomaterials to confine the target reaction to the surface of the working electrode to achieve highly sensitive and selective detection of the target to be measured, so the electrochemical method can well compensate for the shortcomings of the traditional colorimetric method and provide more stable and quantitative detection and analysis [10,11,12]. Some of the commonly used electrochemical detection methods such as electrochemical impedance spectroscopy (EIS) and chronoamperometry (CA) have been applied to POCT of a wide range of analytes [13,14].

Among the microfluidic sensors, paper-based microfluidic sensors have occupied a large part of the market. The preparation of microfluidic channels is achieved by selectively hydrophobizing some areas of the paper base. Due to the special porous structure of the paper, the target analyte can flow along the microfluidic channel without the aid of a pump or other external driving force [15]. In addition to this self-driven liquid transport method, electrophoresis techniques are also applied in lab-on-a-chip to enable the manipulation of micro-particles and small droplets [16,17]. However, the pre-preparation of paper-based sensors is tedious, and due to the strong hydrophilicity of the paper base, the paper-based sensors cannot be cleaned and there will always be reagent loss during the flow. Here, we proposed a PET-based microfluidic sensor. Compared with paper-based sensors, first, the PET material has a certain toughness, which can better protect the electrodes from being damaged by bending. Second, since the PET material is hydrophobic, it can be used multiple times after reasonable cleaning steps, which improves the utilization rate of the material. Finally, due to the presence of liquid surface tension, the natural hydrophobicity of the PET substrate, the analysis reagent can coalesce spontaneously in the test area, saving the need to process the hydrophobic area for paper-based sensors.

## 2. Materials and Methods

### 2.1. Materials

d-(+)-glucose and glucose oxidase (GOx) were purchased from G-CLONE (Beijing, China), 200 Umg^−1^. Potassium ferricyanide (K_3_[Fe(CN)_6_]) and potassium chloride (KCl) were purchased from Yita Biotechnology (Beijing, China). Sulfuric acid was purchased from Yiya Biotechnology. Carbon ink (JC8) and Ag/AgCl ink (JLL20) were purchased from Julong Electronic Technology (Shanghai, China). Polyethylene terephthalate (PET) transparent film (CG7060) was purchased from Amazon. Phosphate buffered solutions (PBS) (10010023) and deionized water (15230162) were purchased from Thermo Fisher Scientific (Ottawa, ON, Canada). All reagents were of analytical grade and used without further purification. The solution of glucose was prepared by dissolving in PBS immediately before each experiment, and the GOx was stored at 4 °C. All solutions were prepared using deionized water.

### 2.2. Design and Fabrication of the Glucose Sensor

**Figure 1:**
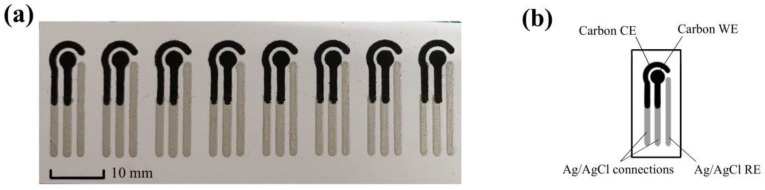
(**a**) Design of the glucose sensor. (**b**) Schematic illustration of a single module with three electrodes.

Figure 1 illustrates the structure of the glucose sensor. Each sensor consists of eight biosensing modules, which are used to quantify the glucose level. Each module includes three different types of electrodes: a working electrode (WE) and counter electrode (CE) made of carbon ink, and a reference electrode (RE) made of Ag/AgCl ink.

The WE and the CE form a circuit so that the current flows smoothly and ensures that the reaction under study occurs at the WE. Since the electrode potentials of the WE and CE are constantly changing during the test, a RE is needed to provide a stable electrode potential during this process to determine the potential of the working electrode [18,19]. Due to the presence of liquid surface tension, the natural hydrophobicity of the PET substrate, and the small size design of the electrodes, we could cover the three electrodes uniformly with a very small amount of sample and reagent (about 3 µL each). Due to the high electrical conductivity of the Ag/AgCl material and its strong adhesion on the PET substrate, we selected the electrodes made of Ag/AgCl ink as contact feet to establish the connection to the electrochemical workstation [20,21].

In terms of dimensions, the shape of the working electrode took the form of a circle with a radius of 1.5 mm, the shape of the auxiliary electrode was a 1/2 circle, and the radius of the test area enclosed by the three electrodes was 3.5 mm. For the microfluidic sensor with a single set of electrodes, the pin spacing between the working electrode, the auxiliary electrode, and the reference electrode was 2.5 mm, the chip length was 25 mm, and the width was 9 mm. The thickness of the PET substrate was 0.2 mm

**Figure 2:**
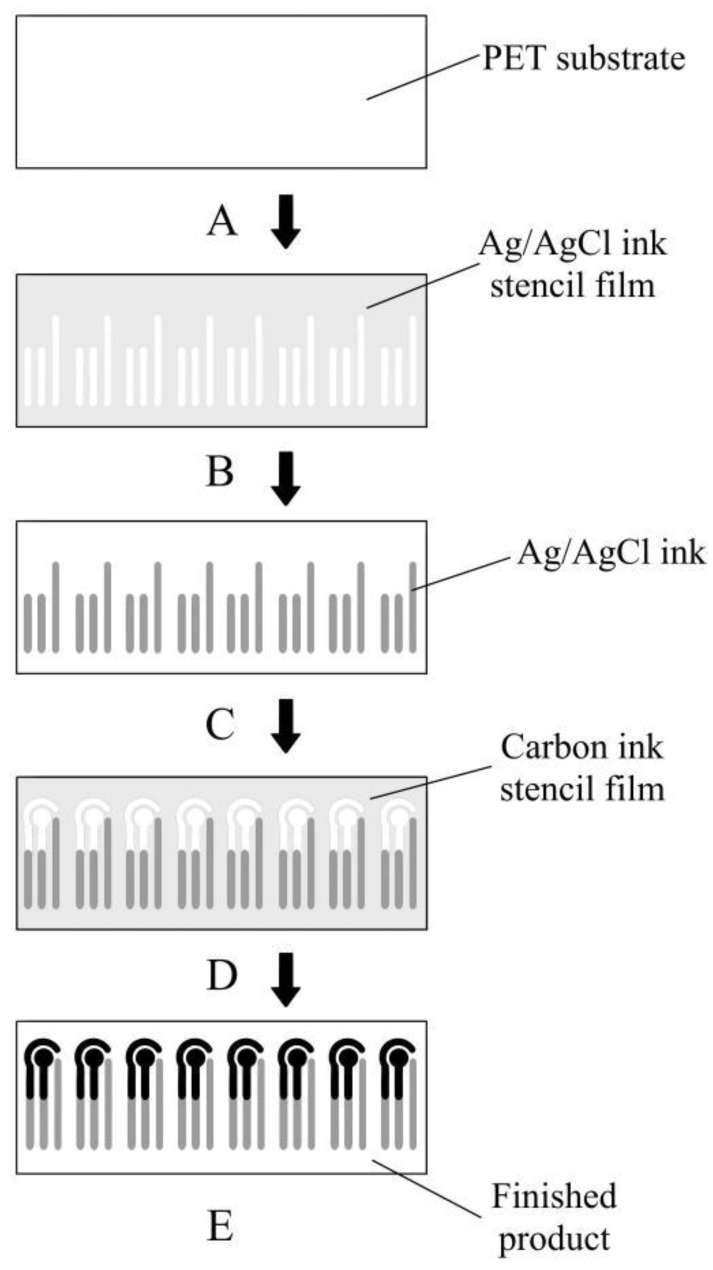
Fabrication process of the glucose sensor.

The electrode is a vital part of an electrochemical sensor. Many methods have been proposed to make electrodes, among which, screen printing is widely used because of its high efficiency and low cost [22,23,24]. Figure 2 illustrates the protocol of glucose sensor fabrication. The first step is to cut the PET substrate to a size that can hold eight sensor modules, then alcohol is used to wipe the surface to remove potential contaminants, thus ensuring a clean surface. The size of each cutout PET substrate needs to be consistent with the size of the stencil film in order to align the carbon and Ag/AgCl electrodes correctly during the printing process. Next, two stencil films for screen printing were processed separately, a carbon ink stencil film for printing WE and CE and an Ag/AgCl stencil film for printing the RE. Laser cutting is used in this process to ensure the surface quality and consistency of the electrode shape of both stencil films. The third step is to print the Ag/AgCl electrode and carbon electrode sequentially. Ag/AgCl electrodes are printed first since they have a stronger adsorption on the PET substrate. If the carbon electrodes are printed first, due to the weak adsorption capacity of the carbon electrodes, there will be a high chance of losing the carbon electrode during the process of printing the silver electrodes and tearing off the stencil film, which will affect the integrity of the electrodes. To dry the printed electrodes, the PET substrate was baked at 50 °C for 60 min after each round of screen printing. This kind of printing method not only improves the production efficiency, but also the finished product, which can be used with a multi-interface electrochemical workstation to perform multiple sets of blood glucose tests simultaneously.

### 2.3. Detection Principles

Currently, glucose sensors are mainly based on enzymatic and non-enzymatic methods. Enzymatic methods are widely used in commercial sensors due to their high specificity for glucose and low cost. Among the enzymatic commercial sensors, two enzymes, glucose oxidase and glucose dehydrogenase (GDH), are the most frequently used. Compared to GDH, GOx is more widely used due to its lower cost, better stability, and higher specificity for glucose. Compared to the optical method, the electrochemical method is based on the electron transfer mechanism during the redox reaction, which usually has higher sensitivity, lower detection lines, and more diverse sensing capabilities. Moreover, used with portable electrochemical instruments, it can be used in clinical tests and POCT. To quantify the blood glucose concentration more precisely, the commonly used oxidant is K_3_[Fe(CN)_6_]. The reaction principle is as follows:

(1) $${\mathrm{Glucose}+2K}_{3}{\left[\mathrm{Fe}(\mathrm{CN}\right)}_{6}{]+H}_{2}O\overset{\mathrm{GOx}}{\to }{\mathrm{Gluconic}\;\mathrm{acid}+2K}_{4}{\left[\mathrm{Fe}(\mathrm{CN}\right)}_{6}]$$

### 2.4. Detection Preparation

After the fabrication of the glucose sensor, the test zone (an area with a radius of about 2 mm from the center of WE) needed to be cleaned with about 10 µL of alcohol and dried at room temperature to remove potential contaminants introduced during the fabrication process. Then, the reaction zone of the WE was loaded with 10 µL of 0.18 M sulfuric acid (diluted in 1 × PBS) and then activated for 3 min with a 1.3 V anodic voltage (against to RE). This activation process creates abundant active functional groups on the WE carbon surface and thus improves the sensitivity of the device [25]. Next, the sensor was washed with 50 µL of deionized water and then with 50 µL of 1 × PBS. The following reagents needed to be configured before the experiment: 200 U mL^−1^ glucose oxidase in 600 mM K_3_[Fe(CN)_6_] and 1 M KCl; 10 mM K_3_[Fe(CN)_6_] (diluted in 1 M KCl); 5 mM, 10 mM, 15 mM, 20 mM d-(+)-glucose (diluted in 1 × PBS).

## 3. Results and Discussions

### 3.1. Electrochemical Characterization of the Glucose Sensor

All test procedures were performed by an electrochemical test platform that consisted of a custom-made electrochemical glucose sensor and a commercial handheld potentiostat (EmStat3 Blue, PalmSens, Guangzhou, China). Since the pin spacing of the glucose sensor was consistent with the design of the potentiostat, it could be directly inserted into the chip slot of the handheld potentiostat for electrochemical signal readout, and the testing data were transmitted through a Bluetooth connection to a smartphone or a computer via USB connection.

Cyclic voltammetry (CV) is one of the most important methods for electroanalytical chemistry research [26,27]. It is often used to test the reversibility of the electrode reaction process, and similarly, we can also characterize the sensor by the test results. [Fe(CN)_6_]^3−^~[Fe(CN)_6_]^4−^ is a typical reversible redox system, and by observing the redox curve of this reaction, we can determine whether this sensor is a reversible electrochemical system. In the experiment, the sensor first needed to be connected to the electrochemical workstation to avoid reagent loss due to the jitter during connection, then 4 µL of 10 mM K_3_[Fe(CN)_6_] (diluted in 1 M KCl) was pipetted onto the test zone of the sensor, and the solution was made sure to cover all three electrodes. In the end, CV waves with scan rates of 50, 100, 150, 200, and 300 mV/s were applied sequentially to observe the experimental results.

**Figure 3:**
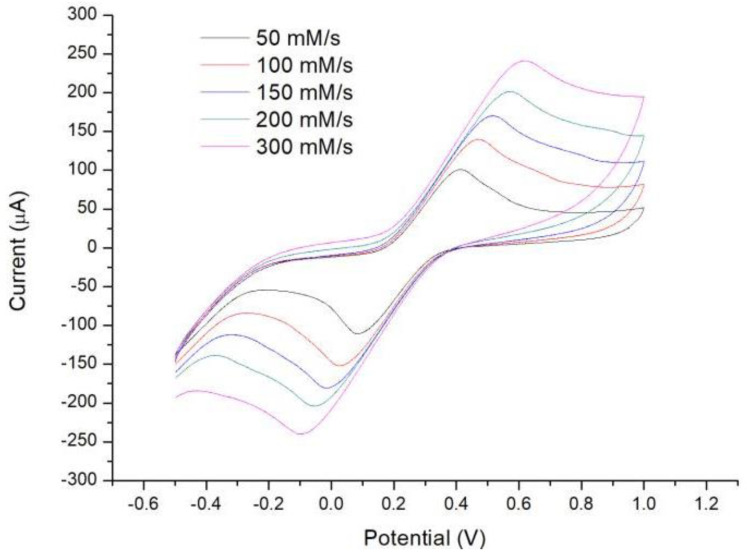
Measurement of cyclic voltammetry (CV) curves at scan rates of 50 mV/s, 100 mV/s, 150 mV/s, 200 mV/s, and 300 mV/s.

**Figure 4:**
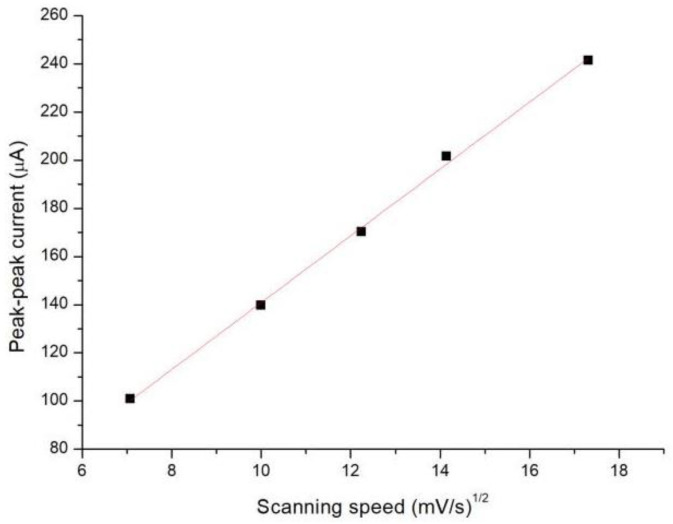
The peak–peak current of the CV curve versus the square root of the scan rate.

As shown in Figure 3, the measured cyclic voltammograms revealed typical reversible electrochemical reactions at all scan rates. The peak–peak current of the cyclic voltammogram was linearly proportional to the square root of the scan rate (Figure 4), further confirming that the glucose sensor is a reversible electrochemical system.

### 3.2. Detection of Glucose

Generally, human blood glucose ranges from 3.9 to 6.1 mM during fasting, beyond which is considered hyperglycemia and below which is considered hypoglycemia. In the blood glucose detection experiment, we used d-(+)-glucose and 1 × PBS to configure a series of solutions to simulate human blood. For each glucose test, the ready-to-use sensor needed to undergo the same pre-processing as the CV measurement.

After the preparation, the sensor was first connected to the electrochemical workstation, and then 3 µL of d-(+)-glucose (diluted in 1 × PBS) and 200 U mL^−1^ glucose oxidase in 600 mM K_3_[Fe(CN)_6_] and 1 M KCl were added separately to the test zone. Ensure that the solutions are in contact with all three electrodes in this step. Among the electrochemical methods, chronoamperometry was chosen to quantify blood glucose levels because it has better accuracy and sensitivity [28]. During the measurement, we applied a step potential of 500 mV to the working electrode and measured the resulting current versus time curve.

**Figure 5:**
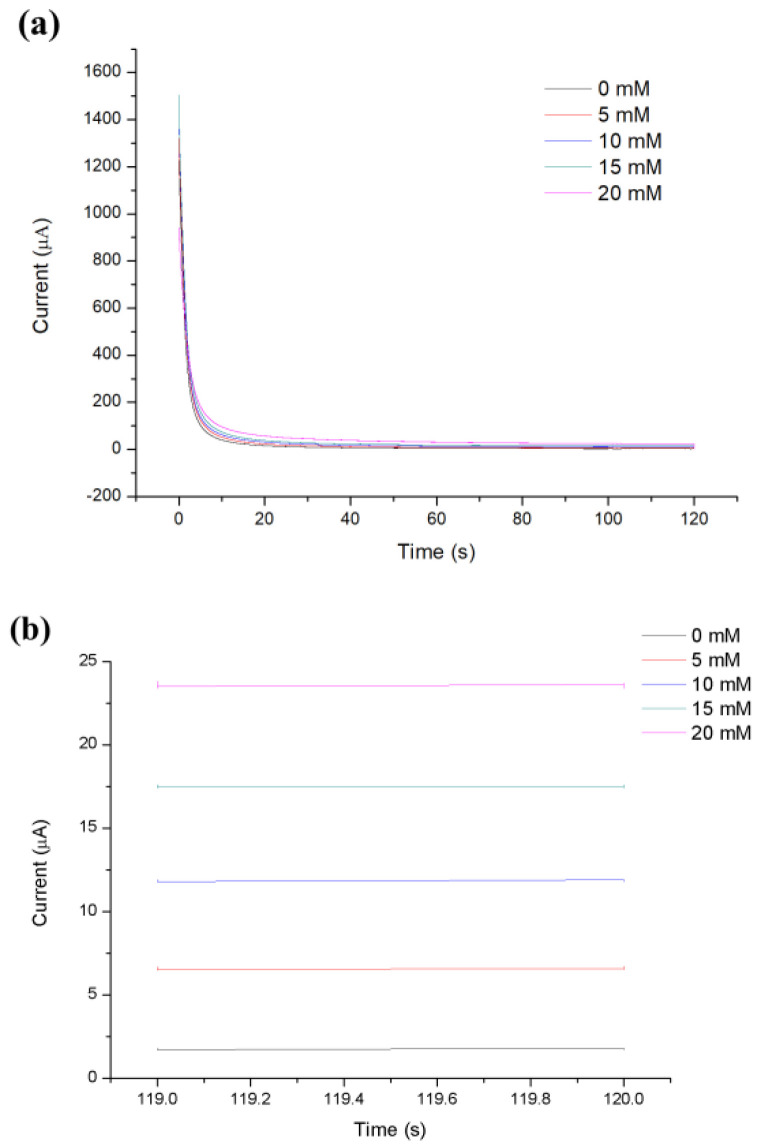
(**a**) Representative CA current curves measured at different concentrations. (**b**) The zoomed-in views of the CA current curves in (**a**) from 119 to 120s.

**Figure 6:**
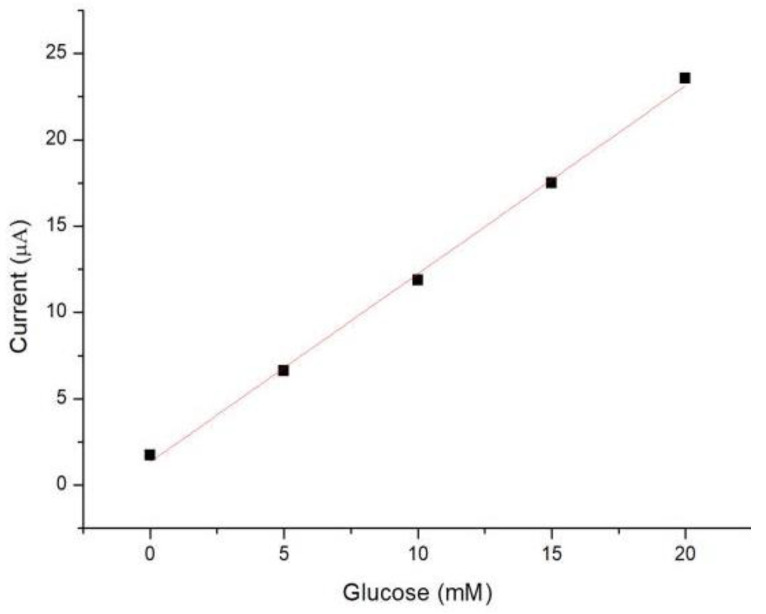
The calibration plot for the measurement of glucose.

To investigate the relationship between glucose concentration and steady-state Faraday current, simulated blood at 0, 5, 10, 15, 20 mM was examined. As shown in Figure 5, the experimental results illustrate that the current reached a steady-state within 120 s after applying the step potential. Therefore, we took the average value of the current measured of 119–120 s as its steady-state Faraday current. As illustrated in Figure 6, the measured Faradaic current was proportional to the concentration of glucose, which can be described by the Cottrell equation.

The limit of detection (LOD) was calculated to be the concentration that generated a current three times the standard deviation of currents measured in zero-concentration simulated blood [29]. The LOD of the glucose sensor in this work was 0.27 mM, which is lower than that of the commercial blood glucose meter (0.83 mM) [30].

## 4. Conclusions

In this work, we developed a PET-based microfluidic sensor and tested it with glucose as the target analyte for relevant measurements and applications. First, we described its design and fabrication method. Then, to verify whether the fabricated sensor could be used for biochemical analysis, it was characterized electrochemically using CV. The potassium ferricyanide cyclic voltammetry experiment was used to scan the solution at five different rates, the results of which illustrated that this sensor is a reversible electrochemical system. For the detection of glucose, enzyme-based electrochemical detection was used in the experiment. A series of simulated blood with concentration gradients were configured and subjected to CA-based electrochemical detection experiments, respectively. Based on the experimental results, it was concluded that the steady-state Faraday current was proportional to the glucose concentration, and a linear fit was performed based on the experimental data, which was well fitted. Finally, the detection limit of this sensor was calculated to be 0.23 mM, which is comparable to existing commercial glucose meters.

## Data Availability

The datasets used and analysed during the current study are available from the corresponding author on reasonable request.
